# Diagnostic and Prognostic Role of WT1 Immunohistochemical Expression in Uterine Carcinoma: A Systematic Review and Meta-Analysis across All Endometrial Carcinoma Histotypes

**DOI:** 10.3390/diagnostics10090637

**Published:** 2020-08-26

**Authors:** Giuseppe Angelico, Angela Santoro, Patrizia Straccia, Frediano Inzani, Federica Cianfrini, Saveria Spadola, Damiano Arciuolo, Michele Valente, Nicoletta D’Alessandris, Antonino Mulè, Gian Franco Zannoni

**Affiliations:** 1 Unità di Gineco-Patologia e Patologia Mammaria, Dipartimento Scienze della Salute della Donna, del Bambino e di Sanità Pubblica, Fondazione Policlinico Universitario A. Gemelli IRCCS, 00168 Roma, Italy; giuangel86@hotmail.it (G.A.); angela.santoro@policlinicogemelli.it (A.S.); stracciapatrizia@libero.it (P.S.); frediano.inzani@policlinicogemelli.it (F.I.); federica.cianfrini@policlinicogemelli.it (F.C.); saveriaspadola@hotmail.it (S.S.); damiano.arciuolo@policlinicogemelli.it (D.A.); dr.valente.m@gmail.com (M.V.); ndalessandris@gmail.com (N.D.); antonino.mule@policlinicogemelli.it (A.M.); 2 Istituto di Anatomia Patologica, Università Cattolica del Sacro Cuore, 00168 Roma, Italy

**Keywords:** endometrial carcinoma, WT1, diagnosis and prognosis, immunohistochemistry, serous carcinoma, carcinosarcoma, clear cell carcinoma, endometrioid carcinoma

## Abstract

**Background**: The diagnostic role of Wilms’ tumor 1 (WT1) is well known in gynaeco-pathological setting, since it is considered a specific marker of serous histotype and adnexal origin. Moreover, its oncogenic role has been recently highlighted in many cancers and it has also been regarded as a promising target antigen for cancer immunotherapy. However, the relationship between its expression and prognostic role in uterine cancer remains unclear. We analyzed the diagnostic and prognostic role of WT1 expression in patients with uterine carcinoma by completing a search using PRISMA (Preferred Reporting Items for Systematic Reviews and Meta-Analyses) guidelines and the PICOS (Participants, Intervention, Comparison, Outcomes, Study Design) model through PubMed, Scopus and Web of Science databases to identify studies that fit our search criteria. The objective of the current meta-analysis was to investigate the diagnostic and prognostic role of WT1 expression in patients with uterine carcinoma. **Materials and Methods**: A literature search was performed of the PubMed, Scopus, and Web of Science databases for English-language studies published from January 2000 to April 2020. Studies were considered eligible if they evaluated the WT1 expression in uterine carcinoma. **Results**: In total, 35 articles were identified that used uterine carcinoma criteria and provided data for 1616 patients. The overall rate of WT1 expression in uterine carcinoma was 25%. The subgroup analysis of uterine cancer types revealed that WT1 was expressed differently among different histotypes (endometrioid, clear cell, serous carcinoma and carcinosarcoma). **Discussion and Conclusions**: The WT1 immunohistochemical expression is not limited to serous histotype and/or ovarian origin. In fact, a significant proportion of endometrial adenocarcinomas can also show WT1 immunoreactivity. Moreover, our study suggests that WT1 may be a potential marker to predict the prognosis of patients with uterine cancer, but more studies are needed to confirm its role in clinical practice.

## 1. Introduction

Endometrial carcinomas (EC) is the most common gynecological malignant neoplasm in industrialized countries and its incidence and mortality has been constantly increasing [1].

To date, it is largely recognized that EC represents a heterogeneous group of diseases with different morphological and molecular features. The first pathogenetic model proposed by Bokhman stratified EC patients in two subgroups: Type I, with high expression of hormonal receptors and a better prognosis; and Type II, which lacks hormone receptors expression and a worse prognosis [2].

A large-scale molecular analysis published in 2013 by the Cancer Genome Atlas (TCGA), defined four molecular categories of endometrial cancer: POLE mutated, hypermutated secondary to microsatellite instability (MSI), low copy number, and high copy number (serous-like) [3].

Despite all these novel pathogenetic and molecular discoveries, EC still carries a high mortality rate and an increase in incidence and mortality is expected over the next few years [4]. Therefore, novel diagnostic and prognostic bio-markers are needed to improve the clinical and therapeutic management of EC patients.

The Wilms’ tumor gene (*WT1*) was first identified in the urogenital system. It encodes a transcription-regulating protein of 52–54 kDa with homology to the prototypic transcription factor family of early growth response genes [5]. It has been shown that WT1 is expressed in various kinds of human cancer including leukemia and myelodysplastic syndrome, brain tumors, neuroblastoma, lung cancer, breast cancer, soft tissue sarcoma as well as in gynecological tumors such as ovarian carcinoma [6,7]. Data from the literature have also revealed that WT1 can promote invasion, migration and metastasis, facilitate angiogenesis and confer drug resistance to cancer cells [5,6].

In the gynecological tract, WT1 is expressed in the surface epithelial cells of the ovaries and fallopian tubes, as well as granulosa cells, myometrium and endometrial stromal cells [8]. Moreover, in gynecological pathology, the immunohistochemical expression of WT1 is useful in the diagnosis of ovarian serous carcinoma (both high grade and low grade histotypes) and is also helpful to distinguish carcinoma of ovarian origin from carcinoma with other primary sites [9]. However, recent papers showed that WT1 immunoexpression can be observed in different histotypes of endometrial carcinoma also suggesting that WT1 may represent a potential prognostic marker in endometrial carcinoma [10].

In the present paper, we conducted a systematic meta-analysis with the aim to elucidate the diagnostic and prognostic role of WT1 immunoexpression in patients with endometrial carcinoma.

## 2. Materials and Methods

### 2.1. Search Strategy

A systematic literature search was performed to identify articles regarding WT1 and prognosis of endometrial carcinoma. Pubmed, Web of Science, and Scopus were used simultaneously, with the combination of terms “WT1 or Wilms’ tumor 1 or Wilms’ tumor gene 1 or Wilms’ tumor protein 1 or Wilms’ tumor suppressor gene 1” and “gynaecological or uterine or endometrial” and “cancer or tumor or neoplasm or carcinoma” (from January 2000 up to April 2020). All articles were initially reviewed by abstract and title browsing to select the relevant reports, which were subjected to further screening.

### 2.2. Study Eligibility

Data retrieved from the studies included the following: author, country, year of publication, follow-up time, total number of patients, mean age, outcome model, overall survival (OS), progression free survival (PFS), relapse/recurrence-free survival (RFS), disease free survival (DFS), WT1 expression in uterine carcinoma, cut-off value of WT1, and stage/grade of tumor according to International Federation of Gynecology and Obstetrics (FIGO) grading and staging system. The language was limited to English only.

### 2.3. Data Extraction

Starting from 140 identified references, 60 duplicates were removed. The first step consisted in an accurate reading of titles and abstracts and the analysis of all the references denoted high intra-rate reliability (98.62% agreement; Cohen *K*: 0.97). A total of 45 references were then retained and a full-text assessment was performed. Finally, 35 references which met the eligibility criteria were retained and included in the current work [10,11,12,13,14,15,16,17,18,19,20,21,22,23,24,25,26,27,28,29,30,31,32,33,34,35,36,37,38,39,40,41,42,43].

The present meta-analysis was conducted according to Guidelines in Preferred Reporting Items for Systematic Reviews and Meta-Analyses (PRISMA) and PICOS (Participants, Intervention, Comparison, Outcomes, Study Design) model. Data from each eligible study were extracted without modification of original data according to the PICOS (Population, Intervention or risk factor, Comparator, Outcomes, Study design) items. “Population” of our study was represented by patients diagnosed with EC. “Intervention” (or risk factor) was the EC group with WT1 expression, assessed by immunohistochemical analysis. “Comparator” was the EC group without WT1 immunohistochemical expression. “Outcomes” were overall survival (OS), progression free survival (PFS), relapse/recurrence-free survival (RFS) and disease free survival (DFS). “Study design” was the study design of the included studies. The PRISMA checklist is shown in Table S1.

### 2.4. Risk of Bias across Studies

**Figure 1:**
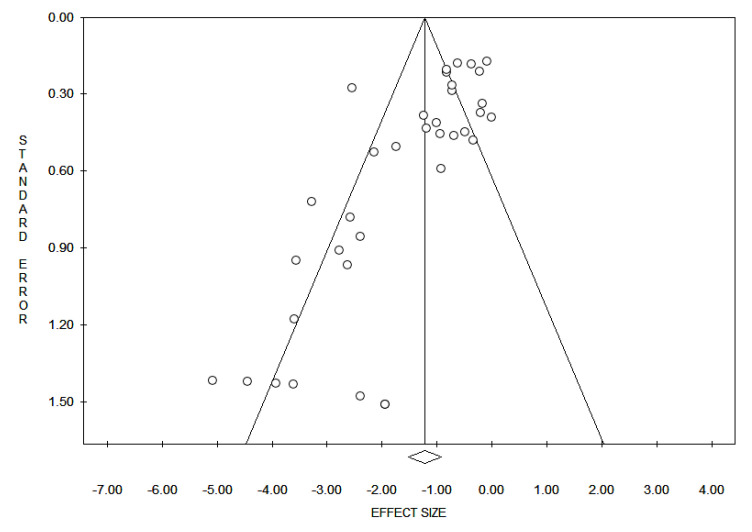
Funnel plot for evaluation of bias across studies: The *x*-axis in the present analysis is the Wilms’ tumor 1 (WT1) expression and the *y*-axis is the standard error. In the absence of bias, a funnel plot should be a symmetrical inverted funnel. In the presence of bias, smaller studies with no expression would be missing, thus creating an asymmetrical funnel. Asymmetry in a funnel plot suggests that there is a systematic difference between larger and smaller studies and/or that there is publication bias.

Reporting bias across studies was evaluated by a graphic diagnostic tool named funnel plot Figure 1. The *x*-axis in the present analysis is the WT1 expression and the *y*-axis is the standard error. In the absence of bias, a funnel plot should be a symmetrical inverted funnel. In the presence of bias, smaller studies with no expression would be missing, thus creating an asymmetrical funnel. Asymmetry in a funnel plot suggests that there is a systematic difference between larger and smaller studies and/or that there is publication bias.

### 2.5. Data Analysis

The rate of WT1 expression in endometrial cancer was calculated for each study included in the meta-analysis, and the results were aggregated using the meta-analytic software ProMeta 2.0 (Internovi, Cesena, Italy). Statistical analysis was performed using MedCalc version 10.2.0.0 (StataCorp LP, College Station, TX, USA) and the GraphPad-Prism 5 software (Graph Pad Software, San Diego, CA, USA). The inverse-variance method was utilized to obtain an overall effect size of the pooled rates of malignancy across studies. Following this, a random-effects model was used as a conservative approach to discriminate the different sources of variation among studies (i.e., within-study variance and between-studies variance) [44].

*Q* and *I*^2^ statistics were then conducted to evaluate heterogeneity across studies [45]. In detail, a significant *Q* value denotes the lack of homogeneity among studies; on the other hand, the proportion of observed variance, which indicates real differences in effect sizes was calculated with *I*^2^ statistics: values of 25%, 50%, and 75% were considered as low, moderate, and high, respectively [46]. Moreover, heterogeneity across study findings was determined using a moderator analysis.

Sensitivity analyses were also performed to determine the stability of study results, computing how the overall rates would change by removing one study at a time. Finally, publication bias analyses were established with two tests: the regression method reported by Egger et al. and the Begg and Mazumdar rank correlation test [46,47,48]. The absence of publication bias is indicated in both tests by non-significant results.

## 3. Results

On the basis of our criteria, the articles that were published between 2000 and 2020 were analyzed and reported in Table 1.

In detail, a total of 35 studies with 1616 patients assessed the role of WT1 expression in patients with uterine carcinoma. The median age was 62.1 years (range 50–71.1). The main characteristics of the studies are reported in Table 1**.** It is worth noting that some studies reported rates of WT1 expression for endometrioid and serous carcinoma (n. 5 studies), for endometrioid and clear cell carcinoma (n. 1 study), for endometrioid, clear cell and serous carcinoma (n. 2 study), and for endometrioid, clear cell carcinoma and carcinosarcoma (n. 1 study), whereas other studies were selective only for one tumor type (n. 26 studies). The shapes of the funnel plots did not reveal evidence of obvious asymmetry (Figure 1).

The shapes of the funnel plots did not reveal evidence of obvious asymmetry.

The results indicated that, in a highly heterogeneous set of 35 studies that compared endometrioid, serous, clear cells carcinoma and carcinosarcoma, the overall rate of WT1 expression was 25% (95% CI = 0.20–0.30; Q = 120.4; I^2^ = 71.7), with *p* < 0.05. Following this, we selected each tumor type and computed the rate of expression.

### 3.1. Analyses of Endometrioid, Serous, Clear Cell Carcinoma and Carcinosarcoma

To provide a comprehensive understanding of the WT1 expression for the single cancer type, additional analyses that included both studies that reported data on the all carcinoma and studies that focused on only a single carcinoma were conducted (Table 1 and Table 2).

Details of the overall rates were tested through moderator analyses. Table 3 illustrates the cut-off values for WT1 in the selected studies.

We also divided all outcomes into two groups including OS, and DFS/RFS/PFS (Table 4). Following this, we presented the main results according to different groups.

### 3.2. Endometrioid Carcinoma

The analyses indicated that the expression of WT1 was 21% (95% CI = 0.16–0.29), in a highly heterogeneous set of 23 studies involving a total of 928 patients (Table 2). The result of publication bias analyses was: Egger test, −3.42; *p* = 0.005; Begg and Mazumdar test, −1.79; *p* = 0.074. For stage assessments, data extracted from studies revealed that tumors classified the FIGO Stage IV had a greater expression of WT1 (28%) than FIGO Stage III (7%) (*p* < 0.05). The combined HR estimate of OS was 27% (95% CI = 0.15–0.44). The combined HR estimate of DFS/RFS/PFS was 24% (95% CI = 0.15–0.35).

### 3.3. Serous Carcinoma

The analyses indicated that the expression of WT1 was 21% (95% CI = 0.14–0.29) in a heterogeneous set of 17 studies involving a total of 289 patients (Table 2). The result of publication bias analyses was: Egger test, −4.01; *p* = 0.001; Begg and Mazumdar test, −1.69; *p* = 0.091. For stage assessments, data extracted from studies revealed that tumors classified the FIGO Stage IV had a greater expression of WT1 (27%) than FIGO Stage III (17%) (*p* < 0.05). The combined HR estimate of OS was 40% (95% CI = 0.19–0.65). The combined HR estimate of DFS/RFS/PFS was 3% (95% CI = 0.00–0.31).

### 3.4. Clear Cell Carcinoma

The analyses indicated that the expression of WT1 was 15% (95% CI = 0.06–0.33); in a set of 6 studies involving a total of 54 patients (Table 2). The result of publication bias analyses was: Egger test, −2.05; *p* = 0.11; Begg and Mazumdar test −0.56; *p* = 0.57. For stage assessments, data extracted from studies revealed that tumors classified the FIGO Stage IV had a greater expression of WT1 (20%) than FIGO Stage III (9%) (*p* < 0.05). The combined HR estimate of OS was 21% (95% CI = 0.08–0.45). The combined HR estimate of DFS/RFS/PFS was 5% (95% CI = 0.01–0.27). Datasets analysis showed that WT1 expression was associated with OS.

### 3.5. Carcinosarcoma

The analyses indicated that the expression of WT1 was 38% (95% CI = 0.33–0.43) in a set of 6 studies involving a total of 240 patients (Table 2). The result of publication bias analyses was: Egger test, 0.34; *p* = 0.75 Begg and Mazumdar test, 0.19; *p* = 0.85. For stage assessments, data extracted from studies revealed that tumors classified the FIGO Stage IV and III had similar levels of WT1 expression (38% and 35%, respectively) (*p* < 0.05). The combined HR estimate of OS was 35% (95% CI = 0.29–0.41). The combined HR estimate of DFS/RFS/PFS was 41% (95% CI = 0.32–0.50). Datasets analysis showed that WT1 expression was associated with DFS/RFS/PFS.

## 4. Discussion

Increasing literature evidence suggests WT1 gene implications in the pathogenesis and prognosis of several solid tumors [5,6,7,8]. Regarding therapeutical strategies, some pilot clinical studies, performed on different types of malignancy, expressing WT1, showed also encouraging results by immunotherapeutic targeting of WT1 [49,50,51,52,53,54]. However, for EC, data concerning safety and tolerability of immunotherapeutic protocols or peptide vaccine with WT1 are limited [55,56,57].

Nevertheless, the clinical-prognostic implications of WT1 expression in endometrial cancer are still controversial. Therefore, to better clarify this issue, we conducted a systematic review and meta-analysis, including all published papers on the WT1 immunohistochemical expression across all histotypes of endometrial carcinoma. The present paper included a total of 35 eligible studies with 52 datasets and 1616 patients for qualitative analysis [10,11,12,13,14,15,16,17,18,19,20,21,22,23,24,25,26,27,28,29,30,31,32,33,34,35,36,37,38,39,40,41,42,43].

Carcinosarcoma and serous histotypes showed the higher rates of WT1 IHC expression (38% and 21% respectively), followed by endometrioid and clear cell histotypes (19% and 15% respectively).

These reported data have important differential diagnostic implications since WT1 IHC is generally believed as the most reliable tool in the distinction between ovarian and endometrial origin of gynecological tumors [8]. In particular, we are aware of the diagnostic challenge encountered in small peritoneal biopsies in cases of peritoneal carcinosis. In this setting immunoreactivity of WT1, particularly if diffuse, could favor tubo-ovarian origin but it is not exclusive of adnexal origin and/or serous histotype. In fact, considering the possibility of WT1 expression also in uterine cancers, difficulty in assigning tumor origin can persist in a minority of cases. In addition, in these contexts, we retain that clinical history, instrumental findings, laboratory markers and a wider immunohistochemistry panel are fundamental to define the correct diagnosis [58,59].

Regarding the impact of WT1 on the cancer patient prognosis most of the scientific studies have shown that positive expression of WT1 was linked with an unfavorable biological behavior.

In an article by Miyoshi et al., a significantly lower disease-free survival rate was observed in breast cancer patients with high levels of WT1 mRNA compared to those with low levels [60]. Similar results were reported in leukemia patients by Inoue et al. In fact, strong WT1 mRNA expression was related to a lower rate of complete remission and worse overall survival [61]. Moreover, the prognostic role of WT1 was also documented in hepatocellular carcinoma patients by Sera et al. In this paper, WT1 protein overexpression, confirmed by Western blotting and immunohistochemistry, represented an independent prognostic factor for disease-free survival [62]. By contrast, Høgdall et al. demonstrated a significantly shorter disease-specific survival in patients affected by ovarian cancer with positive WT1 protein expression [63]. Similarly, Netinatsunthorn et al. reported the prognostic role of WT1 immunohistochemical expression in patients with advanced serous ovarian carcinoma [64].

To date, on the other hand, only few reports are available on the prognostic impact of WT1 expression in endometrial cancer patients. In the present meta-analysis, we observed a worse prognosis in term of OS and DFS/RFS/PFS in EC cases showing strong WT1 expression. In detail, we found that uterine carcinosarcoma with high WT1 expression showed the worst outcome, as also highlighted by Coosemans et al. [18], especially regarding DFS/RFS/PFS. Overall, WT1 expression showed association with OS and DFS/RFS/PFS in endometrioid carcinoma and with OS, especially for serous carcinoma and clear cell carcinoma patients. Moreover, we noted that WT1 showed higher rates of expression in advance FIGO staged cancers (33%) in all histotypes.

It should be noted that there are some limitations to the analysis presented here. First, publication bias should be considered because more positive results tended to be published, thus potentially exaggerating the association between WT1 expression and poor prognosis. Second, there is limited number of studies reporting outcome results, therefore further larger cohorts of EC patients are needed to validate results of the present meta-analysis. Third, we combined DFS/RFS/PFS as a group. Although definitions among DFS/RFS/PFS are not standardized in the majority of our analysis, we consider them equivalent, and the combination can lead a bias.

Finally, we were unable to carry out stratified analysis according to cut-off values of WT1 expression due to numerous methodological variations among selected studies.

## 5. Conclusions

In summary, our study suggests the potential diagnostic and prognostic utility of WT1 in EC patients. Moreover, strong expression of WT1 is associated with poor outcome in this category of affected women. Therefore, we retain that it is important to validate pathological assessment of WT1 expression and its clinical utility by large multicenter prospective studies.

## Figures and Tables

**Table 1:** Characteristics of Included Studies in the Meta-Analysis.

Author	Year	Country	Cancer Type	No of Patients	Age (Mean)	Stage/Grade	Follow up Time (Months)	Outcome	WT1 Positive Expression (%); Cutoff Value
Coosemans, et al. [10]	2008	Belgium	EC SC CCC	24 9 3	NA	I–IV	NA	NA	17/24 (71) 7/9 (77.7) 2/3 (66.6); score ≥ 3
Acs, et al. [11]	2003	USA	EC CCC SC	35 18 16	63.1	I–III	NA	NA	0/35 0/18 10/16; ≥50%
Al-Hussaini, et al. [12]	2003	UK	EC SC	7 25	NA	NA	NA	NA	0/7 (0) 2/25 (8); ≥50%
Atik, et al. [14]	2016	Turkey	EC	50	56	I–III	NA	OS	40/50 (80); score ≥ 3
Baek, et al. [15]	2016	Korea	EC	10	50	I–IV	0–40	OS/DFS	4/10 (40); score ≥ 3
Chen, et al. [16]	2016	Canada	EC CCC	113 17	66	I–IV	NA	DFS	23/113 (18.5); score ≥ 1 0/17 (0)
Chitale, et al. [17]	2005	USA	EC CCC CS	35 12 13	NA	I–III	NA	OS	11/35 (31.4) 2/12(16.6) 7/13(53.8); ≥50% score ≥ 3
Coosemans, et al. [18]	2011	Belgium	CS	71	65	I–IV	≥12 m	OS/PFS	49/71 (69%); score ≥ 20
Dohi, et al. [19]	2009	Japan	EC	70	57.3	I–IV	NA	OS	64/70 (91); ≥50%
Dupont, et al. [20]	2004	USA	EC CCC SC CS	99 4 9 10	65	I–IV	1–241	OS	20/99 (20) 2/4 (50) 3/9 (33.3) 7/10 (70); ≥50%
Egan, et al. [21]	2003	USA	EC SC	39 31	NA	I–III	NA	NA	0/39 (0) 2/31 (6.4); score ≥ 2
Espinosa, et al. [22]	2017	Spain	EC	3	58.6	I–IV	0–48	OS	0/3 (0)
Fadare, et al. [23]	2013	USA	SC	22	NA	I–II	NA	NA	8/22 (36); ≥50%
Franko, et al. [24]	2010	Canada	CS	16	NA	I–IV	NA	NA	13/16 (81); score ≥ 3
Goldstein, et al. [25]	2002	USA	SC	18	NA	NA	NA	NA	0/18 (0)
Guntupalli, et al. [26]	2013	USA	CS	87	68.8	I–IV	1–187	OS	47/87 (54%); score > 21
Hashi, et al. [27]	2003	Japan	SC	13	NA	I–IV	6–142	OS	13/13(100); ≥50%/score ≥ 3
Hedley, et al. [28]	2014	UK	EC	77	69	I–IV	0–56	DFS	34/77 (44); ≥50%
Hirschowit, et al. [29]	2009	UK	SC	34	68.7	NA	NA	NA	4/34 (12); score ≥ 3
Jones, et al. [30]	2019	USA	CS	43	67	I–IV	NA	OS	21/43 (49); score ≥ 3
Kitade, et al. [31]	2019	Japan	SC	5	52.4	I–IV	26–210	NA	0/5 (0)
Lu, et al. [32]	2016	China	SC	3	58	I–III	Median 44	NA	0/3 (0)
Matalka, et al. [33]	2012	Jordan	EC	53	57.8	I–III	NA	NA	2/53 (8.1); score ≥ 3
Nofech-Mozes, et al. [34]	2008	Canada	SC	37	71.1	I–IV	NA	NA	18/37 (48.6); ≥50% score ≥ 3
Nafisi, et al. [35]	2015	Canada	EC SC	23 17	NA	NA	NA	NA	4/23 (17.3) 3/17 (17.6); ≥ 50%
Ohno, et al. [36]	2009	Japan	EC	70	57.3	I–IV	Median 61 m	OS/RFS	31/70 (44%); score ≥ 5
Ruba, et al. [37]	2020	Australia	EC	14	64	I–IV	NA	NA	7/14 (50); >10%
Stanescu, et al. [38]	2014	Romania	EC	79	62	I–III	NA	NA	0/79 (0)
Sumathi, et al. [39]	2004	UK	EC	19	NA	NA	NA	NA	16/19 (84.2); score ≥ 3
Tanvir, et al. [40]	2014	Pakistan	EC	42	63	NA	NA	NA	0/42 (0)
Togami, et al. [41]	2015	Japan	EC SC	29 12	NA	NA	NA	NA	6/29 (21) 0/12 (0); score ≥ 2
Trinh, et al. [42]	2019	Canada	EC SC	37 25	66.8	I–IV	NA	NA	26/37 (70.2) 3/25 (12); ≥50%
Yan, et al. [43]	2013	USA	SC	13	62.2	NA	NA	NA	8/13 (61.5); score ≥ 3

EC: endometrioid carcinoma; CCC: clear cells carcinoma; CS: carcinosarcoma; SC: serous carcinoma; WT1: Wilms’ tumor 1; NA: not available; OS: overall survival; PFS: progression free survival; RFS: relapse/recurrence-free survival; DFS: disease free survival.

**Table 2:** Summary of Meta-Analytic Results.

	K	N	Overall Rate of WT1 Expression (95% CI), %	Q	I^2^
Endometroid Carcinoma	23	985	21 (16−29)	117.07	81.21
Serous Carcinoma	17	307	21 (14−29)	42.3	62.2
Clear Cell Carcinoma	6	59	15 (6−33)	6.99	28.4
Carcinosarcoma	6	240	38 (33−43)	2.31	0.00

K: number of studies; N: total number of patients; CI: confidence interval; I^2^: index for quantifying the degree of heterogeneity; Q: test for heterogeneity; *p* < 0.001.

**Table 3:** Evaluation the cut-off value for Wilms’ tumor 1 (WT1) in the selected studies.

Author	Cancer Type	WT1 Positive Expression (%); Cutoff Value	Cut-Off Value for WT1
Coosemans, et al. [10]	CS	49/71 (69)	A score for each slide was calculated by multiplying the percentage and intensity of positive cells and then categorized as negative (0–20), weak (21–80), moderate (81–180), and strong (181–300).
Acs, et al. [11]	EC CCC SC	0/35 (0) 0/18 (0) 10/16 (62.5)	Score (out of maximum of 300) = sum 1 × percentage of weak, 2 × percentage of moderate, 3 × percentage of strong staining.
Al-Hussaini, et al. [12]	EC SC	0/7 (0) 2/25 (8)	Cases were scored as 0 (totally negative or only occasional scattered positive cells), 1+ (<10% cells positive), 2+ (10–50% of cells positive) or 3+ (>50% of cells positive).
Atik, et al. [14]	EC	40/50 (80)	The total score was calculated by multiplying the intensity and percentage of staining: negative (0), 0–20; weak (1), 21–80; moderate (2), 81–180; and strong (3), 181–300.
Baek, et al. [15]	EC	4/10 (40)	Cases were divided by the intensity of cell staining, given as values of 0, 1, 2, and 3. The percentage stained area was multiplied by this number to calculate the overall score (negative 0–20, weakly positive 21–80, moderately positive 81–180, and strongly positive 181–300).
Chen, et al. [16]	EC CCC	23/113 (18.5) 0/17 (0)	Any staining ≥1% of tumor cells were categorized as positive.
Chitale, et al. [17]	EC CCC CS	11/35 (31.4) 2/12 (16.6) 7/13 (53.8)	The extent of tumor staining was estimated on the basis of numbers of tumor cells stained and graded as follows: Focal, approximately ˂5%; +, 5–25%; ++, 26–50%; +++, 51–75%; and ++++, >75%. Staining in ˂50% of the tumor (+ to ++) was considered heterogeneous staining.
Coosemans, et al. [18]	EC SC CCC	17/24 (71) 7/9 (77.7) 2/3 (66.6)	A scoring system was based on the multiplication of percentage and intensity of positive cells, being negative (0–20), weak (21–80), moderately (81–180) and strong (181–300).
Dohi, et al. [19]	EC	64/70 (91)	Staining intensity was scored as 0 (negative), 1 (weak), 2 (medium), and 3 (strong). The extent of staining was scored as 0 (0%), 1 (1–25%), 2 (26–50%), 3 (51–75%) and 4 (76–100%) according to the percentage of positive staining area in relation to the whole carcinoma area. The sum of the intensity and extent score was used as the final staining score (0–7) for WT1. Tumors having a final staining score of ≥5 were considered to exhibit strong expression.
Dupont, et al. [20]	EC CCC SC CS	20/99 (20) 2/4 (50) 3/9 (33.3) 7/10 (70)	An adaptation of the German immunoreactive score (IRS), negative or weak immunoreactivity (scores 0–3) was considered negative, while moderate or strong immunoreactivity (scores 4–12) was considered positive.
Egan, et al. [21]	EC SC	0/39 (0) 2/31 (6.4)	WT1 was scored on the intensity and localization of the staining of tumor cell nuclei and was graded 0, 1+, 2+, and 3+, representing absent, focal/weak, moderate, and intense expression. Average scores of 0 to 1 were considered negative. Scores of 2 to 3 were interpreted as positive.
Espinosa, et al. [22]	EC	0/3 (0)	Strong expression in tumor cell nuclei.
Fadare, et al. [23]	SC	8/22 (36)	The extent of staining was semi-quantitatively assessed as follows: 0 (0–9%), 1 (10–25%), 2 (26–50%), 3 (51–100%). Any composite score above 0 was considered to be positive.
Franko, et al. [24]	CS	13/16 (81)	Staining intensity was scored as 0 = none, 1 = weak, 2 = intermediate, and 3 = strong and amount as 0 = none, 1 = less than 1%, 2 = 1% to 10%, 3 = 11% to 33%, 4 = 34% to 67% and 5 = more than 67%. Intensity and amount were multiplied to yield a score.
Goldstein, et al. [25]	SC	0/18 (0)	Tumor staining was estimated on the basis of numbers of tumor cells stained and graded as follows: Focal, approximately ˂5%; +, 5–25%; ++, 26–50%; +++, 51–75%; and ++++, >75%.
Guntupalli, et al. [26]	CS	47/87 (54)	WT1 was stratified by absent/low expression (score 0–2), moderate expression (score 3–4), and strong expression (5–6).
Hashi, et al. [27]	SC	13/13 (100)	Staining intensity was scored as 0 = none, 1 = weak, 2 = intermediate, and 3 = strong.
Hedley, et al. [28]	EC	34/77 (44)	Expression of WT1 was considered positive when nuclear staining was identified.
Hirschowit, et al. [29]	SC	4/34 (12)	Immunoreactivity was scored as follows: no reactivity = 0; <10% nuclei positive = 1+; 10–49% positive = 2+; 50–74% positive = 3+; 75–100% positive = 4+.
Jones, et al. [30]	CS	21/43 (49)	Staining intensity was scored as 0 = none, 1 = weak, 2 = intermediate, and 3 = strong. The score ranged to 0 (no immunoreactivity) to 300 (highest immunoreactivity).
Kitade, et al. [31]	SC	0/5 (0)	Staining intensity was scored as 0 = none, 1 = weak, 2 = intermediate, and 3 = strong.
Lu, et al. [32]	SC	0/3 (0)	The percentage of positive cells was scored as follows: − for no immunoreactivity; focally + for 1% to 5%; + for 6% to 25%; ++ for 26% to 50%; +++ for 51% to 75%; ++++ for 76% to 100%.
Matalka, et al. [33]	EC	2/53 (8.1)	WT1 scoring system was based on the multiplication of percentage and intensity of positive cells: negative (0–20), weak (21–80), moderate (81–180), and strong (181–300). Negative or weak immunoreactivity was considered negative, while moderate or strong immunoreactivity was considered positive.
Nofech-Mozes, et al. [34]	SC	18/37 (48.6)	The proportion of positive cells and classified as: negative: 0%; 1+ = 1–25%; 2+ = 25–50% and 3 += strong (>50%).
Nafisi, et al. [35]	EC SC	4/23 (17.3) 3/17 (17.6)	Staining intensity was scored as 0 = none, 1 = weak, 2 = intermediate, and 3 = strong.
Ruba, et al. [37]	EC	7/14 (50)	Positive WT1 expression was defined as moderate to strong nuclear immunoreactivity in >10% of tumor cells.
Stanescu, et al. [38]	EC	0/79 (0)	Immunohistochemical results were either evaluated in a semi-quantitative manner and scored according to the percentages of positively staining cells or in a qualitative manner and appreciated as being positive or negative, paying attention to scoring only tumor cells stained in the appropriate nuclear/membrane position.
Sumathi, et al. [39]	EC	16/19 (84.2)	Cases scored as 0 (negative or only an occasional cell staining), 1+ (˂5% cells positive), 2+ (5% to 25% cells positive), 3+ (26% to 50% cells positive), and 4% (>50% cells positive).
Tanvir, et al. [40]	EC	0/42 (0)	The positive cells were classified as: negative: 0%; 1+ = 1–25%; 2+ = 25–50% and 3+ = strong (>50%).
Togami, et al. [41]	EC SC	6/29 (21) 0/12 (0)	The level of expression was graded according to the percentage of immunoreactive neoplastic cells component as follows: 0, <10%; 1+, 10–25%; 2+, 26–50%; 3+, >50%. Tumors with >10% stained cells were considered positive for expression of that antigen.
Trinh, et al. [42]	EC SC	26/37 (70.2) 3/25 (12)	The positive cells were classified as: negative: 0%; 1+ = 1–25%; 2+ = 25–50% and 3+ = strong (>50%).
Yan, et al. [43]	SC	8/13 (61.5)	The level of expression was graded according to the percentage of immunoreactive neoplastic cells component as follows: 0, <10%; 1+, 10–25%; 2+, 26–50%; 3+, >50%.

EC: endometrioid carcinoma, SC: serous carcinoma, CCC: clear cells carcinoma, CS: carcinosarcoma.

**Table 4:** Combined Hazard Ratio (HR) for OS and PFS in different histotypes of Endometrial Carcinoma.

Histotypes	Combined HR OS	Combined HR PFS
Endometrioid	27%	24%
Serous	40%	3%
Clear Cell	21%	5%
Carcinosarcoma	35%	41%

## Supplementary Materials

The following are available online at https://www.mdpi.com/2075-4418/10/9/637/s1.
