# Supplementary figures and images for: Diagnostic and Prognostic Role of WT1 Immunohistochemical Expression in Uterine Carcinoma: A Systematic Review and Meta-Analysis across All Endometrial Carcinoma Histotypes
Source: Diagnostics (Basel). 2020 Aug 26;10(9):637. doi: 10.3390/diagnostics10090637 (PMC7555656; doi:10.3390/diagnostics10090637)

**Table S1.** Flow diagram of the study selection process.

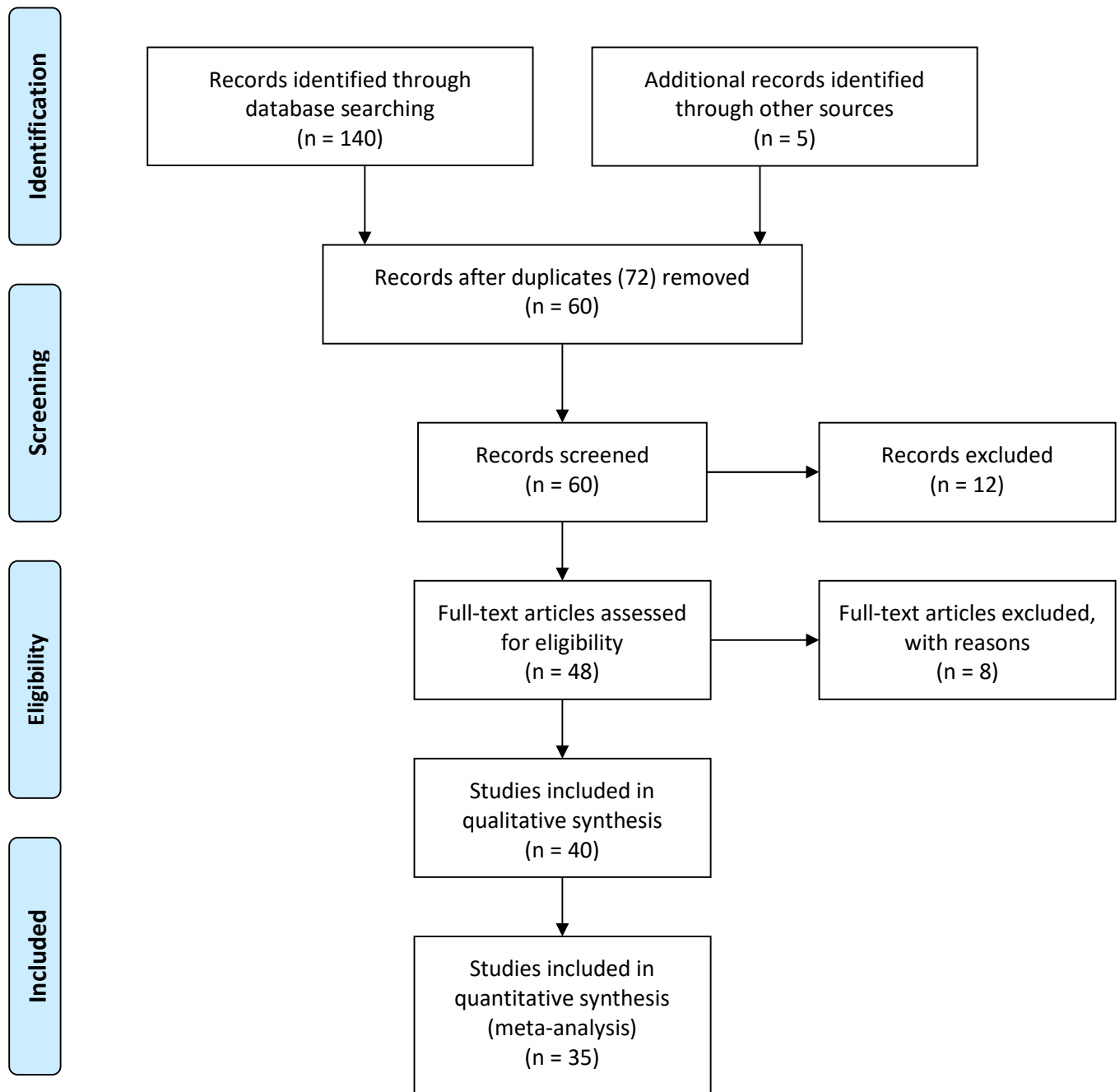

Supplement: Supplementary file 1 [file diagnostics-10-00637-s001.pdf]
